# Evaluation of Immunoprotection Conferred by the Subunit Vaccines of GRA2 and GRA5 against Acute Toxoplasmosis in BALB/c Mice

**DOI:** 10.3389/fmicb.2016.00609

**Published:** 2016-04-27

**Authors:** Xiao T. Ching, Mun Y. Fong, Yee L. Lau

**Affiliations:** Department of Parasitology, Faculty of Medicine, University of Malaya Kuala Lumpur, Malaysia

**Keywords:** *Toxoplasma gondii*, toxoplasmosis, GRA2, GRA5, subunit vaccine

## Abstract

Toxoplasmosis is a foodborne disease caused by *Toxoplasma gondii*, an obligate intracellular parasite. Severe symptoms occur in the immunocompromised patients and pregnant women leading to fatality and abortions respectively. Vaccination development is essential to control the disease. The *T. gondii* dense granule antigen 2 and 5 (GRA2 and GRA5) have been targeted in this study because these proteins are essential to the development of parasitophorous vacuole (PV), a specialized compartment formed within the infected host cell. PV is resistance to host cell endosomes and lysosomes thereby protecting the invaded parasite. Recombinant dense granular proteins, GRA2 (rGRA2) and GRA5 (rGRA5) were cloned, expressed, and purified in *Escherichia coli*, BL21 (DE3) pLysS. The potential of these purified antigens as subunit vaccine candidates against toxoplasmosis were evaluated through subcutaneous injection of BALB/c mice followed by immunological characterization (humoral- and cellular-mediated) and lethal challenge against virulent *T. gondii* RH strain in BALB/c mice. Results obtained demonstrated that rGRA2 and rGRA5 elicited humoral and cellular-mediated immunity in the mice. High level of IgG antibody was produced with the isotype IgG2a/IgG1 ratio of ≈0.87 (*p* < 0.001). Significant increase (*p* < 0.05) in the level of four cytokines (IFN-γ, IL-2, IL-4, and IL-10) was obtained. The antibody and cytokine results suggest that a mix mode of Th1/Th2-immunity was elicited with predominant Th1-immune response inducing partial protection against *T. gondii* acute infection in BALB/c mice. Our findings indicated that both GRA2 and GRA5 are potential candidates for vaccine development against *T. gondii* acute infection.

## Introduction

*Toxoplasma gondii* (*T. gondii*) is a ubiquitous and obligate intracellular protozoan parasite capable of infecting a broad range of warm-blooded hosts (Dubey, 2010), causing a disease known as toxoplasmosis. Toxoplasmosis is a common infection globally distributed affecting up to one-third of the world’s human population (Jackson and Hutchison, 1989). It poses danger to the AIDS patients and pregnant women where fatality and abortions can result respectively. *T. gondii* infection also causes abortions in livestock especially sheep and goats, leading to great economic losses in livestock and food industry (Buxton, 1998).

Infected patients are commonly treated with pyrimethamine, sulphadiazine and spiramycin (during pregnancy) but these drugs are unable to eliminate the parasites completely (Hill and Dubey, 2002; Montoya and Liesenfeld, 2004). The problem of parasites eradication, disease reactivation, toxic effects and emerging drug resistance in parasites makes drug treatment unreliable for long term treatment (Bhopale, 2003; Kur et al., 2009; Innes, 2010). Development of effective vaccines against toxoplasmosis is thus needed to fight against the parasite. To date, Toxovax is the only available vaccine in the market for preventing toxoplasmosis in domestic animals especially sheep and goats. However, this vaccine is not widely acceptable for human use due to the high possibility of regaining the parasite’s pathogenicity (Chen et al., 2009), side effects and high cost of production (Ismael et al., 2003). Production of safe recombinant vaccines is made possible through recombinant DNA technology.

Development of protein-based vaccines are basically safer and more specific in boosting the immune response of the recipients by presenting only selected immunogenic antigens instead of the whole parasite (Schaap et al., 2007). The common route of purified recombinant protein injection is via subcutaneous tissue. Upon injection, the proteins will be taken up by circulating antigen presenting cell (APC) such as macrophage. The proteins will then be processed into peptide-MHC class II complex within APC before being presented on the cell surface to CD4^+^ helper T cells, stimulating humoral-mediated immunity (Th2) resulting in antibody production. Difficulties in generating Th1 immunity can be overcome by formulating the recombinant proteins with appropriate adjuvants as they play important role in directing the desired Th1/Th2 profiles (Kur et al., 2009; Bruna-Romero et al., 2012). For example, formulation of alum (Th2 inducer) and IL-12 (Th1 inducer) result in a strong Th1 activity (Schaap et al., 2007). Other adjuvants that are commonly used in subcutaneous injection are Freund’s complete adjuvant (FCA), Freund’s incomplete adjuvant (FIA), liposomes and IL-12.

*Toxoplasma gondii* infection begins when the tachyzoites invade host cells. Uncontrolled replication of the tachyzoites leads to rupturing of the infected cells thereby releasing new parasites to invade neighboring cells. The parasite remains protected within a parasitophorous vacuole (PV), a specialized compartment formed within the infected host cell during and after invasion. Dense granules (GRAs) are *T. gondii* specialized secretory organelles involved in PV development whereby the antigens helped in the maturation and modification of both PV and PV membrane (Nam, 2009). GRAs are the major components of both vacuole surrounding tachyzoites and encysted bradyzoites (Capron and Dessaint, 1988; Cesbron-Delauw and Capron, 1993) which have been identified as potential vaccines (Scorza et al., 2003; Hiszczynska-Sawicka et al., 2011; Sun et al., 2011).

GRA2 contributes to the formation of intravacuolar network in PV, allowing proteins and nutrients transportation to nourish the parasites while GRA5 helps to inhibit apoptosis of the infected cells thereby protecting the parasites during cell invasion (Feng et al., 2002; Nam, 2009). Both GRA2 and GRA5 are expressed throughout the whole intermediate host life cycle of *T. gondii* thus preventing stage-limited protection against toxoplasmosis (Tilley et al., 1997; Zhou et al., 2007).

Several studies had been conducted on the evaluation of multi-component vaccine candidate incorporating GRA2 or GRA5 with other potential genes against toxoplasmosis (Zhou et al., 2007; Igarashi et al., 2008a; Xue et al., 2008; Liu et al., 2009). However, limited number of study had been performed on these two target genes as single antigen vaccine especially GRA5. The only report on rGRA2 expressed in *Escherichia coli* as single subunit vaccine candidate investigated its efficacy against chronic toxoplasmosis based on the *T. gondii* brain cysts counts (Golkar et al., 2007). Nevertheless, protective effect conferred by the same antigen against lethal parasitic infection of type I virulent strain has not been reported yet. In this study, recombinant GRA2 and GRA5 proteins were subjected to mice immunization study as single antigen subunit vaccine candidates against acute *T. gondii* infection in BALB/c mice.

## Materials and Methods

### Mice

Six- to eight-week old female BALB/c mice were purchased from Monash University Sunway Campus. The mice were maintained in a pathogen free environment and were fed ad lib with commercial food pellets and water.

### Ethics Statement

This study was carried out in strict accordance with the recommendations in the Guide for the Care and Use of Laboratory Animals of the National Institutes of Health. The protocol was approved by the Institutional Animal Care and Use Committee (IACUC) of the University of Malaya, Faculty of Medicine (Permit Number: 2014-06-03/PARA/R/CXT).

### Parasites Propagation and Harvest

*Toxoplasma gondii* tachyzoites of the virulent wild-type RH strain were provided by the Department of Parasitology, University of Malaya, Kuala Lumpur, Malaysia. They were propagated *in vitro* involving infection of Human Foreskin Fibroblast (HFF) cells. DMEM complete medium was replaced with parasite infection medium 12–16 h pre-infection when the growth of HFF cells reached 80–90% confluence. Freshly isolated or frozen parasites were washed with sterile phosphate-buffered saline (PBS) and resuspended in parasite infection medium before infecting the HFF cells. After 24 h of incubation in the CO_2_ incubator, the infected cells were replaced with new parasite infection medium in order to remove free floating tachyzoites. Incubation was continued until the lysis of the infected cells triggered by the actively replicating tachyzoites thereby releasing them into the medium.

During lysis of the infected HFF cells, erupted tachyzoites and the infected cells were harvested with a cell scraper (TPP, USA). The entire cell suspension was transferred to a syringe attached to a 25 gage needle which was placed into a 50 ml polypropylene tube beforehand. The cell suspension was forced to pass through the needle by the plunger to ensure the release of tachyzoites from the intact infected cells. The act was repeated twice followed by two times of washing with sterile PBS whereby the cell suspension was centrifuged at 1,000 × *g* for 15 min between each wash. After the last wash, the pellet obtained was resuspended in sterile PBS before filtering through 3 μm polycarbonate membrane (Merck, USA). The filtrate containing only the parasites was sedimented and resuspended in sterile PBS prior to usage.

### Recombinant GRA2 and GRA5 Proteins Production

Recombinant GRA2 and GRA5 plasmids were constructed and the sequences were verified. Heterologous expression of the positive clones was performed in BL21 (DE3) pLysS followed by affinity purification. Identity of the purified recombinant proteins were validated through MALDI-TOF MS analysis before subjected to vaccination study. The entire procedures were described thoroughly in our previous report (Ching et al., 2015).

### Immunization Regime

Six- to eight-week old female inbred BALB/c mice were divided into four immunization groups with 13 mice in each group; two negative controls: PBS and pRSET B, two protein groups: rGRA2 and rGRA5. Three injections were administered subcutaneously with final protein dose of 10 μg at 2 weeks intervals. Injection samples were emulsified with complete/incomplete Freud’s adjuvant (C/IFA) at 1:1 ratio before immunizing the mice. Blood (50–100 μl) were collected from the immunized mice through tail-bleeding on day 0, 14, 28, and 42.

### Evaluation of Humoral Response

Mice serum samples harvested were analyzed by western blot assay (Ching et al., 2015) and in-house ELISA against the purified recombinant proteins to detect the presence of antigen-specific IgG antibodies and to determine antibody titers.

### IgG Titer and Subclass Determination

The 96-well flat bottom microplate was coated overnight at 4°C with 10 μg/ml rGRA2 or rGRA5 diluted in 100 μl coating buffer. The antigen solutions were aspirated and the wells were washed three times with 0.05% PBS-T after overnight incubation. The subsequent incubation steps were all carried out at 37°C. Non-specific binding sites of the wells were blocked by incubation with 200 μl of 10% blocking buffer for 1 h. The blocking buffer was then aspirated and the plate was washed thrice followed by incubation with 100 μl of serially diluted mice sera (control and vaccinated) for another 1 h to determine the optimal working dilution. The plate was washed again in the same way. Bound antigen-specific IgG was detected through incubation with 100 μl of diluted HRP-conjugated goat anti-mouse IgG (1:2000) for 1 h. The plate was washed five times and the enzymatic reaction was developed by addition of 100 μl 3,3′,5,5′-Tetramethylbenzidine (TMB), a chromogenic substrate and was incubated for 10–15 min at RT. The reaction was eventually stopped with 2 M of sulphuric acid and the absorbance was measured at 450 nm with microplate reader. Primary and secondary antibodies were diluted in 10% blocking buffer. All samples were run in triplicates. Vaccinated mice sera were considered positive if the mean optical density (OD) of triplicate determinations was greater than the cut-off limit of the negative control groups; cut-off = mean OD + 2(standard deviation). The entire steps were repeated for IgG subclass determination assay whereby different secondary antibodies involved were HRP-conjugated goat anti-mouse IgG1 and IgG2a.

### *In Vitro* Splenocyte Proliferation Assay

Three mice per group were euthanized with CO_2_ and spleens were harvested aseptically two weeks after final immunization. Single cell suspension was prepared by mashing the spleen over a 70 μm cell strainer and washed with 10 ml RPMI complete medium (CM) before subjected to centrifugation. All centrifugation steps were performed at 1,500 rpm for 10 min. Cell pellet obtained was resuspended with 5 ml of ACK lysis buffer and incubated for 5 min at RT. Five minute later; the cell suspension was washed with 20 ml CM and was centrifuged again. This step was repeated with 10 ml CM. Cell pellet obtained was eventually resuspended in 10 ml CM. Splenocytes were cultured in 96-well flat bottom microplate with cell density of 2 × 10^5^ cells/well in triplicates. The cells were induced with culture medium alone (negative control), 10 μg/ml rGRA2 or rGRA5 or 5 μg/ml con A (positive control) before incubated at 37°C in a 5% CO_2_ incubator for 24, 72, and 96 h.

Splenocytes proliferation was analyzed with MTT Cell Proliferation Kit at 72 h post-incubation according to the instruction’s manual. Briefly, MTT labeling reagents was added into each well of cells and were incubated for 4 h followed by incubation overnight with 100 μl of Solubilization solution. The plate was read at 570 nm with microplate reader the next day.

$$Stimulation\,\;index\left(S1\right)=\frac{mean\,\;OD_{570}\,\;values\,\;of\,\;stimulated\,\;cells}{mean\,\;OD_{570}\,\;values\,\;of\,\;unstimulated\,\;cells}$$

### Cytokine Assays

Splenocytes cultured and incubated at different time point (24, 72, and 96 h) were subjected to centrifugation at 2,000 × *g* for 20 min. Culture supernatants were collected for various cytokine assays such as IFN-γ, IL-2, IL-4 and IL-10 assays. These assays were performed in accordance with the instruction’s manuals.

### Mice Challenge

The remaining vaccinated and control mice were subjected to lethal parasitic challenge study through intraperitoneal injection of 1 × 10^3^ live tachyzoites of *T. gondii* virulent RH strain. Mortality rate of the mice were monitored and recorded twice daily whereby the infected mice were observed for end-point criteria that is when they were heavily infected, showing symptoms of sluggish movement, hunched back posture, ruffled and thinning hair coat as well as obvious reduced food and water consumption. The heavily infected mice that reached end-point criteria were humanely killed by exposure to a gradually increasing concentration of carbon dioxide (CO_2_) inside a closed chamber.

### Statistical Analysis

Significance levels of the differences between groups of mice were analyzed through Student’s *t*-test or analysis of variance (ANOVA). *P* < 0.05 indicates statistical significance. The survival rate was calculated based on χ^2^ (chi-square) test while the survival graph was drawn based on Kaplan–Meier method (Kaplan and Meier, 1958).

## Results

### IgG Antibody Detection

**FIGURE 1:**
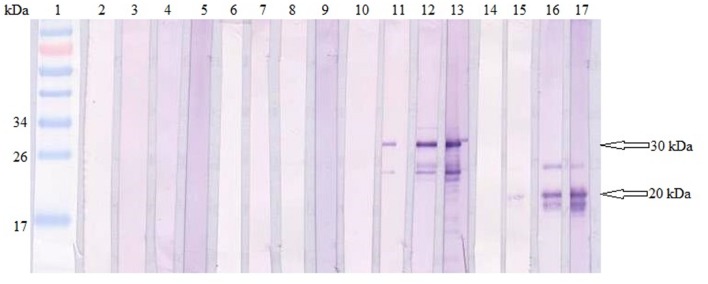
**Qualitative detections of total specific anti-GRAs IgG antibodies in mice sera.** Western blots of purified recombinant proteins (rGRA2 or/and rGRA5) with sera of the immunized mice. Lane 1 contained PageRuler Prestained Protein Ladder. Lane 2–5 were incubated with sera of PBS-injected mice, lane 6–9 were incubated with sera of pRSET B-injected mice, lane 10–13 were incubated with sera of rGRA2-immunized mice and lane 14–17 were incubated with sera of rGRA5-immunized mice. The 4 sera represented sera collected at week 0, 2, 4, and 6 post-prime injections. The 30 kDa purified rGRA2 and 20 kDa purified rGRA5 were first detected at week 2 (lane 11 and 15 respectively) followed by an increase in the band intensity at week 4 and 6. No bands were observed in the mice sera of the two control groups.

**FIGURE 2:**
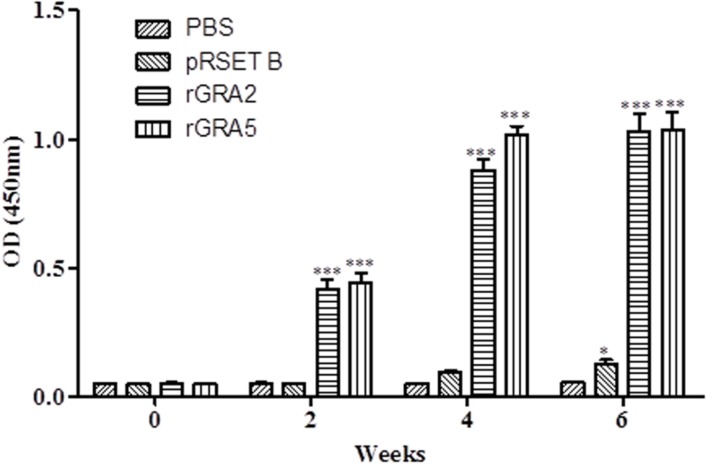
**Quantitative detections of total specific anti-GRAs IgG antibodies in mice sera.** Total anti-GRAs IgG antibodies in mice sera were detected and evaluated by ELISA. Sera were collected from each mouse group one day before each immunization. Data are expressed as mean OD_450_ ± SD (*n* = 3). Statistical differences are represented by ^∗^ (significant; *p* < 0.05) and ^∗∗∗^ (highly significant; *p* < 0.001) in comparison with the control groups (PBS or pRSET B).

Total specific anti-GRA2 and anti-GRA5 IgG antibodies were detected in the sera collected from the mice immunized with rGRA2 and rGRA5 respectively through SDS-PAGE/WB (**Figure 1**) and ELISA (**Figure 2**; **Table 1**) against purified recombinant proteins. Faint protein bands with target size of 30 and 20 kDa were observed at week 2 after prime injection of mice with rGRA2 and rGRA5 respectively as shown in **Figure 1**. The intensity increased at week 4 and 6 following first and second booster injections. However, antibody detection was not observed in Native-PAGE/WB (data not shown). Meanwhile, **Figure 2** also showed the same phenomena whereby significantly higher levels of IgG antibodies were observed in the recombinant protein-vaccinated groups compared to two control groups (*p* < 0.001) and the level gradually elevated with successive immunizations. There was no statistical difference between the two control groups at week 2 and 4 (*p* > 0.05). However, IgG level in pRSET B-injected group was found significantly higher than that of PBS-injected group (*p* < 0.05) at week 6 post-prime injection. The level of IgG antibodies in mice sera collected from rGRA5-vaccinated group was significantly higher than that of rGRA2-vaccinated group (*p* < 0.001) at week 4 but both groups reached highest level 2 weeks after the last injection (week 6) without statistical difference (*p* > 0.05). These results indicated that both recombinant GRA2 and GRA5 proteins are immunogenic and capable of stimulating significantly strong humoral immune response in the respective vaccinated mice compared to the negative control groups.

**Table 1:** Specific anti-GRAs IgG antibody profile in sera collected from the immunized BALB/c mice 2 weeks after last injection.

Group (*n* = 3)	OD_450_		
	
	IgG	IgG1	IgG2a
rGRA2	1.0330 ± 0.0645^∗∗∗^	2.9760 ± 0.2025^∗∗∗^	2.5850 ± 0.0303^∗∗∗^
rGRA5	1.0370 ± 0.0679^∗∗∗^	3.0770 ± 0.0528^∗∗∗^	2.6820 ± 0.0768^∗∗∗^
pRSET B	0.1267 ± 0.0170^∗^	0.0801 ± 0.0020	0.0686 ± 0.0033
PBS	0.0540 ± 0.0007	0.1256 ± 0.0060	0.0717 ± 0.0078


*Data are expressed as mean ± SD. Statistical differences are represented by ^∗^ (significant; *p* < 0.05) and ^∗∗∗^ (highly significant; *p* < 0.001) in comparison with the control groups (PBS or pRSET B). Each group consisted of three mice.*

### IgG Titer Determination

Antibody titer is a quantitative measurement of the amount of antibody capable of recognizing the respective epitope. It is usually expressed as reciprocal of the highest dilution with an OD450 greater than the positive cut-off value of IgG (mean + 2 SD) relative to the control mice sera at the same dilution. Antibody titer of both the anti-GRA2 and anti-GRA5 IgG was determined to range from 1:409,600 to 1:819,200 by ELISA.

### IgG Antibody Isotypes Determination

**FIGURE 3:**
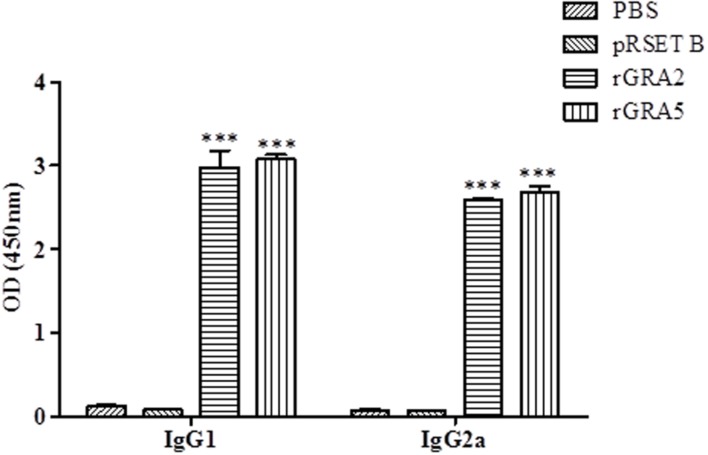
**IgG isotypes determination in the immunized BALB/c mice sera.** Polyclonal antibody isotypes (IgG1 and IgG2a) in the immunized mice sera were determined by ELISA. Sera from each mouse group were collected 2 weeks after the last injection. Data are expressed as mean OD_450_ ± SD (*n* = 3). Statistical difference is represented by ^∗∗∗^ (highly significant; *p* < 0.001) in comparison with the control groups (PBS or pRSET B).

Polyclonal antibody isotypes (IgG1 and IgG2a) in the immunized BALB/c mice sera were further assessed by ELISA in order to identify type of immunity being triggered. The levels of specific anti-GRAs IgG1 and IgG2a being produced are depicted in **Figure 3** and tabulated in **Table 1**. Generally, the level of IgG isotypes present in the sera of the two vaccinated mice groups were highly significantly greater than that of the two control groups (*p* < 0.001). No statistical difference was observed between the two control groups (*p* > 0.05) and also between the two vaccinated groups (*p* > 0.05). On top of that, it was shown that high levels of two IgG isotypes were detected in all the rGRA2- and rGRA5-immunized mice sera, with slightly higher level of IgG1 compared to IgG2a, giving rise to an IgG2a/IgG1 ratio of <1 (≈0.87). The result obtained indicated that both Th1 and Th2 immune responses were driven in all the vaccinated mice.

### *In Vitro* Splenocytes Proliferation Assay

**FIGURE 4:**
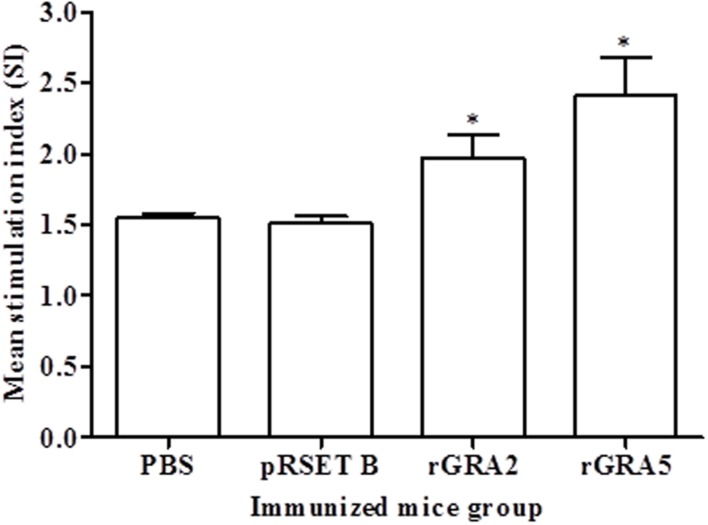
***In vitro* splenocytes proliferation response in mice.** Spleen lymphocytes were harvested from mice immunized with rGRA2, rGRA5, pRSET B and PBS 2 weeks after last injection. The splenocytes were cultured and stimulated with the respective recombinant proteins. Proliferative response was measured by MTT assay. Data are expressed as mean stimulation index (SI) ± SD (*n* = 3). Statistical difference is represented by ^∗^(*p* < 0.05) in comparison with the control groups (PBS or pRSET B).

Antigen-specific proliferative response of splenocytes from each mice group to rGRA2 or/and rGRA5 stimulus was determined using MTT assay and represented by the SI value as illustrated in **Figure 4** and **Table 2**. Generally, significantly higher SI value was observed in the recombinant protein-vaccinated groups compared to the control groups (*p* < 0.05). On top of that, splenocytes from rGRA5-vaccinated mice had significantly stronger proliferation compared to that of rGRA2-vaccinated mice in response to their respective stimulus (*p* < 0.05). Nevertheless, there was no statistical difference between the two control groups (*p* > 0.05). Meanwhile, SI value for all mice groups had comparable levels in response to the mitogen conA. These results indicated that T lymphocytes of the vaccinated mice were successfully stimulated.

**Table 2:** Characterization of cellular-mediated immunity in the vaccinated mice.

Group (*n* = 3)	Proliferation (SI)	Cytokine level (pg/ml)			
		
		IFN-γ	IL-2	IL-4	IL-10
rGRA2	1.973 ± 0.1589^∗^	4645 ± 1032^∗^	1527 ± 247^∗^	9.989 ± 2.231	121.6 ± 43.94^∗^
rGRA5	2.414 ± 0.2674^∗^	4724 ± 372.5^∗^	1232 ± 95.07^∗^	25.46 ± 11.98^∗^	178.3 ± 27.71^∗^
pRSET B	1.514 ± 0.0447	197.4 ± 61.45	146 ± 42.01	Undetectable	61.55 ± 16.18
PBS	1.549 ± 0.0345	142.7 ± 77.15	123 ± 13.67	Undetectable	26.98 ± 8.667


*SI stands for stimulation index. IFN-γ activity was assayed at 96 h, IL-2 and IL-4 activities were assayed at 24 h, and IL-10 activity was assayed at 72 h. Undetectable IL-4 level was observed in the stimulated splenocytes culture supernatant of the negative control mice groups. Data are expressed as mean ± SD (*n* = 3). Statistical difference is represented by ^∗^ (*p* < 0.05) in comparison with the control groups (PBS or pRSET B).*

### Cytokine Production Assay

**FIGURE 5:**
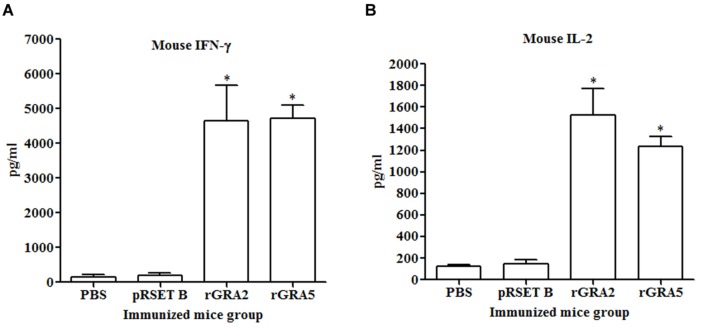
**(A,B)** IFN-γ and IL-2 production by the stimulated splenocytes of the immunized mice. Culture supernatants from the antigen-stimulated immunized mice splenocytes were harvested at 96 and 24 h post-incubation for the evaluation of **(A)** IFN-γ and **(B)** IL-2 production respectively via ELISA. Data are expressed as mean ± SD (*n* = 3). Statistical difference is represented by ^∗^(*p* < 0.05) in comparison with the control groups (PBS or pRSET B).

**FIGURE 6:**
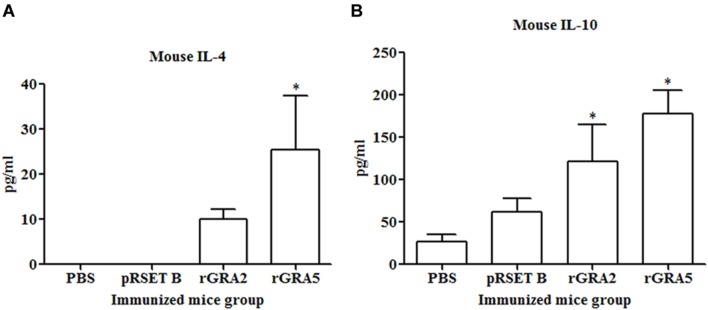
**(A,B)** IL-4 and IL-10 production by the stimulated splenocytes of the immunized mice. Culture supernatants from the antigen-stimulated immunized mice splenocytes were harvested at 24 and 72 h post-incubation for the evaluation of **(A)** IL-4 and **(B)** IL-10 production respectively via ELISA. Data are expressed as mean ± SD (*n* = 3). Statistical difference is represented by ^∗^(*p* < 0.05) in comparison with the control groups (PBS or pRSET B).

Results obtained showed that splenocytes of the vaccinated mice produced significantly higher level of IFN-γ and IL-2 compared to the control groups (*p* < 0.05) as demonstrated in **Figure 5** and **Table 2**. No statistical difference was observed between two vaccinated groups (*p* > 0.05) and between two control groups (*p* > 0.05). In contrast, relatively low levels of IL-4 and IL-10 were released by the stimulated splenocytes of the mice immunized with rGRA2 and rGRA5 (**Figure 6**; **Table 2**). Undetectable level of IL-4 was observed in the control groups (**Table 2**). However, IL-10 level produced by splenocytes from the recombinant protein-vaccinated mice was significantly higher compared to PBS and pRSET B-injected mice (*p* < 0.05). Production of huge amount of IFN-γ and IL-2 and relatively low level of IL-4 and IL-10 indicated that Th1 immune response was favored in the vaccinated mice.

### Protective Efficacy of Recombinant Protein Vaccination in BALB/c Mice

**FIGURE 7:**
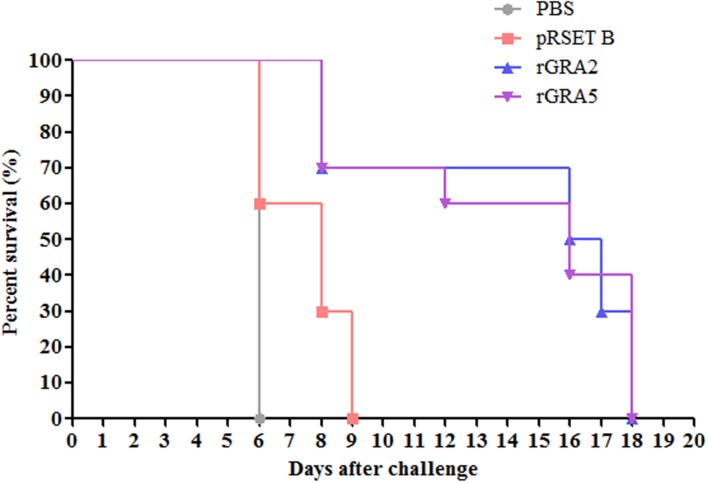
**Survival rate of the immunized mice.** All four groups of the immunized mice (PBS, pRSET B, rGRA2, rGRA5) were subjected to lethal challenge with 1000 live tachyzoites of *T. gondii* virulent RH strain 2 weeks after the last immunization. Mice immunized with rGRA2 and rGRA5 exhibited a significant increase in the survival days (median survival of 16.5 and 16 days respectively) in comparison to the control mice injected with PBS and pRSET B (median survival of 6 and 8 days respectively). Each group consisted of 10 mice.

Protective efficacy of recombinant GRA2 and GRA5 proteins in the immunized BALB/c mice were evaluated against lethal challenge with *T. gondii*. The survival rates of the four challenged mice groups were illustrated in **Figure 7**. It was shown that the two vaccinated mice groups had significantly prolonged survival rates as compared to the two control mice groups (PBS and pRSET B) (*p* < 0.05). All PBS- and pRSET B-injected mice succumbed to the parasite infection on day 6 (median survival of 6 days) and day 9 (median survival of 8 days) respectively. Meanwhile, rGRA2- and rGRA5-immunized mice died within 8–18 days post-infection with the median survival of 16.5 and 16 days respectively. Although all the immunized mice died on day 18, but these two subunit vaccines were successfully demonstrated to increase the survival rates of the vaccinated BALB/c mice against *T. gondii* infection.

## Discussion

Subunit vaccination is generally known for its efficacy in inducing humoral immune responses against extracellular pathogens through antibody generation which is favored by T helper 2 (Th2) related responses. Th1 and Th2 responses are characterized by differences in cytokine production and by antibody isotype (Snapper and Paul, 1987; Gazzinelli et al., 1991; Germann et al., 1995; Bessieres et al., 1997). However, *T. gondii* is an intracellular parasite, thus an immune response mediated by CD4^+^ Th1 and CD8^+^ cytotoxic T cells are the main components required to combat this parasitic infection (Denkers and Gazzinelli, 1998). Immunization using animal model with recombinant expressed protein alone is weakly immunogenic (Sloat et al., 2010) and often elicited a mixed Th1/Th2-like response with higher tendency of IgG1 isotype production, driving predominantly Th2-like response (Echeverria et al., 2006; Dziadek et al., 2009, 2012; Sun et al., 2014). Therefore, addition of Th1-directing adjuvant such as Complete Freund’s adjuvant (CFA) was used in this study. This was to enhance the immunogenicity of the subunit vaccine as well as directing the immune response toward Th1.

Immunogenicity and protective efficacy of several *T. gondii* recombinant antigens produced in bacteria have also been performed against *Toxoplasma* infection in experimental mouse models (Martin et al., 2004; Dziadek et al., 2009, 2012). It was reported that alum adjuvant-formulated rGRA4 was a potential multi-antigen vaccination candidate against chronic *T. gondii* infection either alone or in combination with rROP2 which were both produced in pQE expression vector. It protected the vaccinated C57BL/6 and C3H mice against challenge with ME49 strain through brain cyst reduction (Martin et al., 2004). Meanwhile, vaccination of C3H/HeJ mice with rROP2 and rROP4 which were expressed in pH is vector has been shown to elicit mixed Th1/Th2-type immune response with specific IL-2 production. These two antigens conferred partial protection against challenge with DX strain (low virulent) with 46% brain cysts reduction. It was also reported that combination of the same rROP2 and rROP4 antigens with either rGRA4 or rSAG1 triggered both humoral (generation of high levels of IgG1 and IgG2a) and cellular- (secretion of IFN-γ and IL-2) associated immunity. The brain cysts loads in the vaccinated BALB/c mice were greatly decreased (84 and 77% reduction respectively) compared to PBS-injected mice (Dziadek et al., 2009, 2012).

In this study, immunization of BALB/c mice with rGRA2 and rGRA5 emulsified with complete/incomplete Freund’s adjuvant (C/IFA) successfully triggered both humoral and cellular mixed Th1/Th2-like immune responses, predominantly Th1 in the vaccinated mice. The triggered immune response eventually prolonged the mice’s survival rates against lethal challenge with the virulent RH strain of *T. gondii*. These results confirmed the antigenicity and immunogenicity of the two recombinant proteins. However, Freund’s adjuvant is not suitable for use in humans due to safety concerns (Stills, 2005).

Analysis of IgG antibody through SDS-PAGE/WB and ELISA indicated that anti-GRA2 and anti-GRA5 IgG antibody was produced in the immunized mice 2 weeks after prime injection. The antibody levels increased with successive immunization where antibody titres ranged from 1:409,600 to 1:819200 at 6 weeks post-prime injection. Meanwhile, anti-GRAs IgG antibody detection was not observed in Native-PAGE/WB probably due to protein folding which hinders the B-cell epitope. Besides, relatively high levels of IgG1 and IgG2a were detected in the immunized mice serum. The levels of these two antibody isotypes are almost similar with slightly higher IgG1 than that of IgG2a. Production of IgG1 is Th2 related, while IgG2a is associated with Th1-driven immunity (Germann et al., 1995).

CD4^+^ Th1 cell populations are involved in B cells activation and subclass-switched antibody production, whereby its absence would lead to increased susceptibility to *T. gondii* (Harris et al., 2001; Johnson and Sayles, 2002). Induction of humoral immune response plays an essential role in the resistance against *Toxoplasma* infection in which most of the B cell-deficient mice survived from the infection post-treatment with immune serum (Frenkel and Taylor, 1982). Significant protection elicited through intraperitoneal injection of monoclonal anti-*T. gondii* surface antigen antibody into mice against moderately and highly virulent *Toxoplasma* infection further supports the importance of humoral immunity in fighting toxoplasmosis (Johnson et al., 1983). Other than conferring resistance to *T. gondii* acute infection and controlling its chronic infection, humoral immunity has been demonstrated to be important in protecting rodents against other protozoa parasites as well, such as *Plasmodium berghei yoelii* (Weinbaum et al., 1976) and *Trypanosoma cruzi* (Rodriguez et al., 1981).

A study on the protective and resisting roles of B cells response against lethal challenge with virulent strains of *T. gondii* demonstrated that antigen-specific antibody inhibited host cell active invasion by blocking the tachyzoites directly, preventing them from attaching to the host cell and thus restricting parasite propagation (Sayles et al., 2000). On the other hand, antibody-coated tachyzoites could be destroyed by phagocytic cells such as macrophage through passive phagocytosis (Sibley et al., 1993). Positive correlation was observed between the high titers of two IgG isotypes; IgG1 and IgG2a and the levels of phagocytosis which eventually protected immunized mice against *S. pneumonia* (Lefeber et al., 2003).

Earlier findings reported that monoclonal antibody against *T. gondii* surface antigens successfully blocked tachyzoites invasion and *in vitro* propagation as compared to monoclonal antibody against antigens of *T. gondii* secretory organelles with little or no effect on invasion (Johnson et al., 1983; Grimwood and Smith, 1996). However, another study showed that phagocytic cell (macrophage) invasion of *T. gondii* was inhibited by monoclonal anti-GRA2 in the presence of complement (Cha et al., 2001). At the same time, the monoclonal antibody partially protected mice against RH strain tachyzoite infection mediated by complement-dependent effector mechanism (Sayles et al., 2000; Cha et al., 2001). This highlights the protective role played by specific antibody.

Apart from humoral immunity, cell-mediated immunity is a major protective response against intracellular *T. gondii*. The cell-mediated immunity response is through specific T lymphocytes activation (CD4^+^ and CD8^+^), especially Th1 response coupled with IFN-γ production (Suzuki and Remington, 1988; Gazzinelli et al., 1991; Parker et al., 1991; Sibley et al., 1993). Interferon-gamma-mediated cytotoxic T lymphocyte (CTL) response restraints the propagation and spreading of the parasite by impeding the growth of actively dividing tachyzoites (acute phase) and limiting reactivation of the encysted bradyzoites (Pfefferkorn, 1984; Pfefferkorn et al., 1986; Suzuki et al., 1988; Dillon et al., 1992; Zheng et al., 2013).

In this study, stimulated T-lymphocytes in the spleen cells of rGRA2- and rGRA5-immunized mice proliferated significantly. Interferon-gamma and IL-2 were two pro-inflammatory cytokines that were secreted in large amount suggesting that CD4^+^ Th1 and CD8^+^ cytotoxic T-cells were being triggered. In addition, IL-4 and IL-10 were also produced but at relatively low levels which are associated with CD4^+^ Th2 cells induction. Besides fighting against *T. gondii*, these two anti-inflammatory cytokines play a vital role in balancing and reducing the deleterious inflammatory effect, especially of IFN-γ (Snapper and Paul, 1987; Gazzinelli et al., 1991; Bessieres et al., 1997).

Humoral and cellular immune responses are interrelated and synergistic instead of acting alone to mount protection against any pathogen. Activated cytokine-secreting Th cells are involved in the stimulation of antibody-producing B cells as well as determining the switch of antibody isotype, either IgG1 or IgG2a in T-cell dependent immunity (Snapper and Paul, 1987; Germann et al., 1995). Th1-related IFN-γ induces IgG2a generation and expression of the respective FcR1 on mouse macrophage (equivalent to human monocyte FcR), suppressing IgG1 synthesis at the same time. In other words, Th1 is responsible for macrophage activation through stimulation of pro-inflammatory cytokines (IFN-γ, TNF-α, IL-2) and IgG2a antibody generation. The protective role of IFN-γ and IgG2a has been demonstrated through opsonisation, complement-mediated cell lysis and antibody-dependent cellular cytotoxicity (ADCC) (Johnson et al., 1985; Snapper and Paul, 1987; Petersen et al., 1998). On the other hand, Th2-related IL-4, which is also known as B cell stimulatory factor-1 (BSF-1), possesses an antagonistic effect to IFN-γ whereby it enhances IgG1 production and FcR2 expression on mouse macrophage (equivalent to natural killer cell FcR in human) but suppresses IgG2a production (Perussia et al., 1983; Snapper and Paul, 1987). Th2 stimulates development of anti-inflammatory cytokines (IL-4, IL-5, IL-6, and IL-10) and IgG1 antibody which leads to down regulation of macrophage activity (Petersen et al., 1998).

A study demonstrated that sterile protective immunity to toxoplasmosis is possible if strong humoral and cellular immune responses are successfully elicited and acting synergistically whereby the survival rates of the infected mice will not significantly increase in the absence of either antibody production or T cell immunity (Frenkel and Taylor, 1982). Therefore, it is deduced that the increase in the median survival time of *T. gondii*-challenged mice from 6–8 days (non-immunized mice; PBS and pRSET B-injected) to 16–16.5 days (immunized mice; rGRA5 and rGRA2-injected) in this study might be resulted from the interaction between antibody-producing B lymphocytes and cytokine-producing T lymphocytes (Th1 and Th2).

The delayed onset of death observed in rGRA2 and rGRA5-immunized mice might be due to the interplay between Th1 and Th2-driven responses as findings have shown that Th1-related cytokine primarily IFN-γ causes early mortality whereas Th2-related IL-4 and IL-10 diminish short-term fatality by down regulating Th1 response and thus reducing the severe inflammatory effect provoked by IFN-γ at the early acute phase of toxoplasmosis (Roberts et al., 1996; Neyer et al., 1997; Petersen et al., 1998). An increase in survival rate and decrease in necrosis of the small intestine was observed in C57BL/6 mice treated with monoclonal anti-IFN-γ antibody (Liesenfeld et al., 1996). It has also been determined that secretion of IFN-γ is directly proportional to the mortality rate of the infected mice (McLeod et al., 1989). IL-4-deficient mice have higher susceptibility toward acute *Toxoplasma* infection compared to the wild type due to excessive IFN-γ secretion. In contrast, development of necrotic lesions with free living tachyzoites has been seen in wild-type mice but not in IL-4-deficient mice (Roberts et al., 1996). One of the negative regulatory effects of IL-10 is to suppress macrophage killing activity mediated by IFN-γ (Sibley et al., 1993). Infected IL-10-depleted mice died during acute *Toxoplasma* infection with relatively high levels of IFN-γ and IL-12 detected in their serum. These mice were believed to have succumbed to lethal immunopathology instead of parasitic infection as there was no sign of significant *T. gondii* propagation (Gazzinelli et al., 1996).

The overall results obtained in the present study are in agreement with the results of previous studies. The recombinant antigens triggered strong humoral and cellular Th1-dominating immune response by up-regulating the development of antigen-specific IgG antibody (IgG2a) and Th1-related cytokines (IFN-γ and IL-2) (Golkar et al., 2007; Zhou et al., 2007). Monophosphoryl lipid A (MPL) adjuvant-formulated rGRA2 had been shown to reduce brain cysts formation significantly in the immunized CBA/J mice either alone or mixed with rGRA6, thereby protecting against chronic *T. gondii* infection (Golkar et al., 2007). Immunization of BALB/c mice with multi-antigenic protein vaccine containing SAG1-GRA2 expressed in yeast host successfully increased survival time of the vaccinated mice up to 15 days against lethal challenge with *T. gondii* RH strain (acute infection) (Zhou et al., 2007).

Protective efficacy of GRA5 subunit vaccine against chronic toxoplasmosis has been indicated by intranasal immunization in combination with rGRA7 and rROP2 adjuvanted with cholera toxin by reducing brain cyst formation of VEG strain by 58.3% in BALB/c mice (Igarashi et al., 2008b). Nevertheless, this is thus far the first report of evaluation of the immunity elicited by GRA5 as a single-antigenic subunit vaccine candidate against acute toxoplasmosis in the mouse model.

## Conclusion

Subcutaneous injection of mice with subunit vaccines rGRA2 and rGRA5 successfully triggered humoral and cellular responses which resulted in partial protection to the vaccinated mice against parasitic lethal challenge. A combination of Th1/Th2-related responses primarily Th1 was obtained with significant increased production of IgG2a, IFN-γ, IL-2 and IgG1 but relatively low level of IL-4 and IL-10. The encouraging findings obtained in this study provide a basis for further investigation into the development of a recombinant multi-antigenic candidate using combination of GRA2-GRA5 for immunization against *T. gondii* infection.

## Author Contributions

YL and MY conceived and designed the study, and critically revised the manuscript. XT performed the experiments, analyzed the data and drafted the manuscript. All authors read and approved the final manuscript.

## Conflict of Interest Statement

The authors declare that the research was conducted in the absence of any commercial or financial relationships that could be construed as a potential conflict of interest.
